# Supplementary material for: Fungi found in Mediterranean and North Sea sponges: how specific are they?
Source: PeerJ. 2017 Sep 6;5:e3722. doi: 10.7717/peerj.3722 (PMC5591636; doi:10.7717/peerj.3722)
Supplement: Table S1 — Sample codes indicate H. panicea (P1-P3), H. xena (X1-X3), S. massa (M1-M3), P. ficiformis (F1-F3), A. aerophoba (A1-A3), A. damicornis (D1-D3), A. verrucosa (V1-V3) and sea water (WNS & WMS). [file peerj-05-3722-s001.docx]

| **Sample** | **Adapter A** | **Barcode** | **Forward Primer (FF390)** | **ERS Accession Number** |
| --- | --- | --- | --- | --- |
| WNS | CCATCTCATCCCTGCGTGTCTCCGACTCAG | AACGGCTT | CGATAACGAACGAGACCT | ERS225559 |
| P1 | CCATCTCATCCCTGCGTGTCTCCGACTCAG | AACCTTGG | CGATAACGAACGAGACCT | ERS225550 |
| P2 | CCATCTCATCCCTGCGTGTCTCCGACTCAG | AACGAACG | CGATAACGAACGAGACCT | ERS225551 |
| P3 | CCATCTCATCCCTGCGTGTCTCCGACTCAG | AACGAAGC | CGATAACGAACGAGACCT | ERS225552 |
| X1 | CCATCTCATCCCTGCGTGTCTCCGACTCAG | AACGATCC | CGATAACGAACGAGACCT | ERS225553 |
| X2 | CCATCTCATCCCTGCGTGTCTCCGACTCAG | AACGATGG | CGATAACGAACGAGACCT | ERS225554 |
| X3 | CCATCTCATCCCTGCGTGTCTCCGACTCAG | AACGCCAT | CGATAACGAACGAGACCT | ERS225555 |
| M1 | CCATCTCATCCCTGCGTGTCTCCGACTCAG | AACGTACC | CGATAACGAACGAGACCT | ERS225556 |
| M2 | CCATCTCATCCCTGCGTGTCTCCGACTCAG | AACGTAGG | CGATAACGAACGAGACCT | ERS225557 |
| M3 | CCATCTCATCCCTGCGTGTCTCCGACTCAG | AACGTTCG | CGATAACGAACGAGACCT | ERS225558 |
| WMS | CCATCTCATCCCTGCGTGTCTCCGACTCAG | AAGCTAGG | CGATAACGAACGAGACCT | ERS225571 |
| F1 | CCATCTCATCCCTGCGTGTCTCCGACTCAG | AAGCATCC | CGATAACGAACGAGACCT | ERS225560 |
| F2 | CCATCTCATCCCTGCGTGTCTCCGACTCAG | AAGCATGG | CGATAACGAACGAGACCT | ERS225561 |
| F3 | CCATCTCATCCCTGCGTGTCTCCGACTCAG | AAGCCGAA | CGATAACGAACGAGACCT | ERS225562 |
| A1 | CCATCTCATCCCTGCGTGTCTCCGACTCAG | AACGTTGC | CGATAACGAACGAGACCT | ERS225563 |
| A2 | CCATCTCATCCCTGCGTGTCTCCGACTCAG | AAGCAACG | CGATAACGAACGAGACCT | ERS225564 |
| A3 | CCATCTCATCCCTGCGTGTCTCCGACTCAG | AAGCAAGC | CGATAACGAACGAGACCT | ERS225575 |
| D1 | CCATCTCATCCCTGCGTGTCTCCGACTCAG | AAGCCGTT | CGATAACGAACGAGACCT | ERS225565 |
| D2 | CCATCTCATCCCTGCGTGTCTCCGACTCAG | AAGCGCAA | CGATAACGAACGAGACCT | ERS225566 |
| D3 | CCATCTCATCCCTGCGTGTCTCCGACTCAG | AAGCGCTT | CGATAACGAACGAGACCT | ERS225567 |
| V1 | CCATCTCATCCCTGCGTGTCTCCGACTCAG | AAGCGGAT | CGATAACGAACGAGACCT | ERS225568 |
| V2 | CCATCTCATCCCTGCGTGTCTCCGACTCAG | AAGCGGTA | CGATAACGAACGAGACCT | ERS225569 |
| V3 | CCATCTCATCCCTGCGTGTCTCCGACTCAG | AAGCTACC | CGATAACGAACGAGACCT | ERS225570 |

**Table S1.** List of barcode sequences and ERS accession numbers used in this study. Sample codes indicate *H. panicea* (P1-P3), *H. xena* (X1-X3), *S. massa* (M1-M3), *P. ficiformis* (F1-F3), *A. aerophoba* (A1-A3), *A. damicornis* (D1-D3), *A. verrucosa* (V1-V3) and sea water (WNS & WMS).
