# Supplementary material for: Fungi found in Mediterranean and North Sea sponges: how specific are they?
Source: PeerJ. 2017 Sep 6;5:e3722. doi: 10.7717/peerj.3722 (PMC5591636; doi:10.7717/peerj.3722)
Supplement: Table S2 [file peerj-05-3722-s002.docx]

**Table S2**. Blastn query at 11-Jan-2017 of selected representative OTUs with highest number of sponge (Porifera) reads from pyrosequencing data from each of the sponge species (and also each of the individual samples belonging to that species) against the non-redundant nucleotide database.
