# Supplementary figures and images for: Fungi found in Mediterranean and North Sea sponges: how specific are they?
Source: PeerJ. 2017 Sep 6;5:e3722. doi: 10.7717/peerj.3722 (PMC5591636; doi:10.7717/peerj.3722)

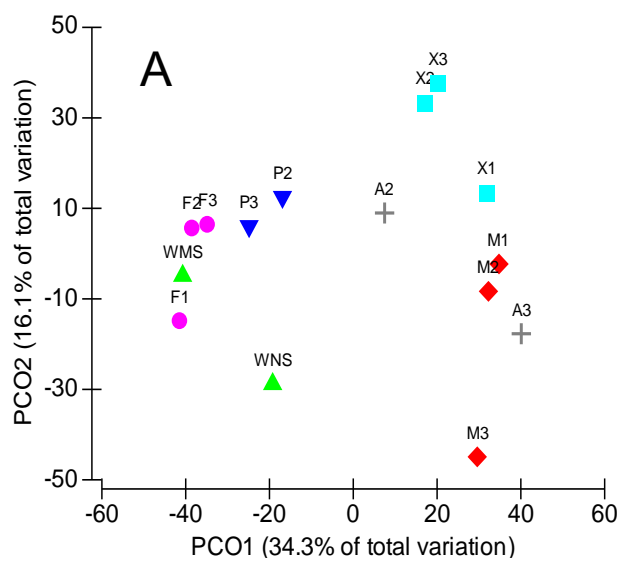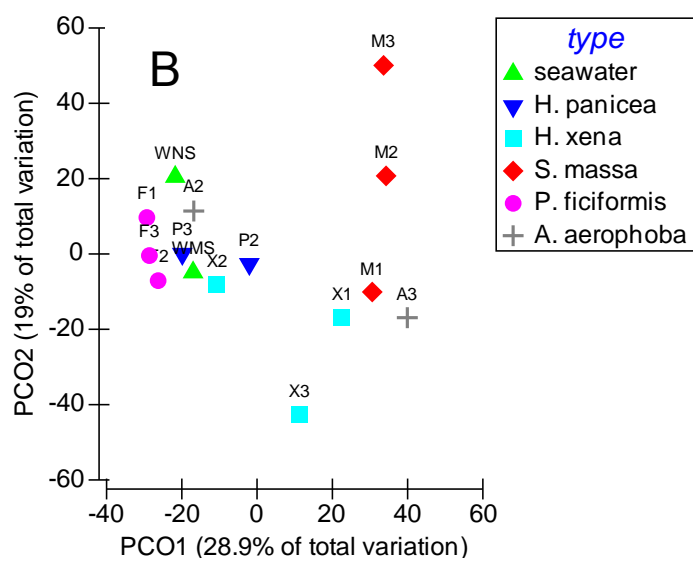

Supplement: Figure S1 — Only samples with more than 10 fungal reads were included. Plots are based on square root transformed relative abundance data of OTUs (A) or presence-absence data (B). The percentage of total variation that is explained by each PCo axis is given in the parentheses. [file peerj-05-3722-s004.pdf]
